# Core acupoint selection strategies and multifactorial analysis for acute musculoskeletal pain

**DOI:** 10.3389/fmed.2026.1805633

**Published:** 2026-05-07

**Authors:** Yide Fang, Ran Xiao, Yanlan Kang, Cheng Chen, Zhenghang Bian, Ruirui Xue, Bing Shu, Jinhai Xu, Wen Mo

**Affiliations:** 1 Department of Orthopedics, Longhua Hospital, Shanghai University of Traditional Chinese Medicine, Shanghai, China; 2 Spine Disease Research Institute, Shanghai Research Institute of Traditional Chinese Medicine, Shanghai, China; 3 Institute for Medical Philosophy and Future Artificial Intelligence, Baotou, Inner Mongolia, China

**Keywords:** acupuncture, acute musculoskeletal pain, bibliometrics, motion style acupuncture treatment, single-point needling, sprain, visualization analysis

## Abstract

**Background:**

This study aims to synthesize research hotspots in acute musculoskeletal pain, elucidate strategies for core acupoint selection and their related factors, and provide evidence-based references for clinical practice.

**Methods:**

A comprehensive literature search was conducted across eight Chinese and English databases up to October 15, 2025, to identify clinical studies utilizing 1–3 acupoints for treatment of acute musculoskeletal pain (AMP). CiteSpace was applied for bibliometric and visualization analyses, extracting core elements for frequency statistics and generating visualization pathway maps based on pain locations. Concurrently, multivariate logistic regression was performed to further explore associations between core elements and specific pain locations. A total of 438 studies were included, including 404 in Chinese and 34 in English.

**Results:**

Chinese publications demonstrated a fluctuating trend over time, while the number of English studies gradually increased after 2010. Keyword clustering showed that motion style acupuncture treatment (MSAT) has become a recent research focus in both Chinese and English literature. Descriptive analysis suggested that Chinese and English studies show similarities in diagnosis and lesion location distribution, with distal acupoints predominating and body acupuncture being the most common technique. Chinese studies emphasized hand acupuncture, while English studies more frequently applied wrist-ankle acupuncture. Perpendicular needle insertion was the most prevalent needling angle. Single-point prescriptions were widely adopted. Chinese studies tended to integrate exercise treatment. Regression analysis demonstrated significant correlations between ankle pain, low back pain, distal acupoint selection, hand acupuncture, and scalp acupuncture with exercise therapy. Although the English study sample is smaller, distal acupoint selection was consistently correlated with exercise. Further analysis revealed that low back pain and shoulder pain primarily employed distal acupoints, while thigh, knee, and upper limb pain favored proximal acupoints. Low back pain and ankle pain were more often treated in combination with exercise therapy.

**Conclusion:**

This study indicated that treating acute musculoskeletal pain with a small number of core acupoints is feasible, and motion style acupuncture is a key technical approach, especially beneficial for acute lower back and ankle injuries.

**Systematic review registration:**

https://www.crd.york.ac.uk/PROSPERO/view/CRD420251155446, identifier CRD420251155446.

## 1 Introduction

Musculoskeletal disorders are a leading cause of pain and disability worldwide, affecting over 1.7 billion individuals. Acute musculoskeletal pain (AMP) refers to pain caused by injury to muscles, bones, joints, ligaments, or soft tissues, lasting less than 4 weeks (1, 2). Common etiologies include sprains, strains, soft tissue injuries, whiplashes, and contusions (3, 4). Without timely and appropriate management, acute pain may develop into chronic pain syndromes (5). Therefore, early and effective interventions are essential. AMP is prevalent across all age groups and is one of the leading complaints in emergency and outpatient clinics. Current treatment mainly relies on opioid and non-opioid analgesics. However, the side effects and dependence risks of opioids have prompted recommendations for multimodal analgesia strategies, emphasizing the need for evidence-based non-pharmacological alternative therapies (2, 6). Acupuncture is a fundamental component of traditional Chinese medicine (TCM). Based on meridian syndrome differentiation, it involves the insertion of needles into specific acupoints to treat diseases and alleviate symptoms (7). As a safe, economical, and evidence-based therapy, acupuncture has gained growing recognition for its potential in managing acute pain (5).

Acupuncture prescriptions targeting specific points demonstrate certain feasibility in treating AMP. Shuzhong Gao has discussed this concept, and multiple clinical studies have demonstrated that rapid symptom relief in certain specific conditions, especially pain, can be achieved through the use of single acupoints (8–11). These studies hold practical relevance in emergency pain management, where rapid efficacy, minimal acupoint usage, and short treatment durations are crucial (12). Research indicates that patients treated with emergency acupuncture report not only significant pain relief but also improvements in anxiety, stress, and sleep quality (13). At the neurophysiological mechanistic level, acupuncture may induce neuroplastic changes in brain, particularly in modulating connectivity and function in motor-related cortical regions (14, 15). Different acupoints activate distinct brain regions associated with specific functions, thus enabling targeted therapy efficacy. The primary motor cortex (M1) plays a direct and critical role in motor recovery, motor control, and neuroplastic remodeling (16–18). The increased excitability of M1 not only promotes the recovery of motor function but also supports the neurocognitive foundation for adapting to new motor pattern (19). Despite its widespread use in musculoskeletal pain, there is still a lack of standardization of acupoint selection strategies. Many randomized controlled trials (RCTs) employ combinations of five or more acupoints, complicating the evaluation of specific acupoints efficacy and thus limiting the replicability and clinical applicability. In contrast, the approach of selecting fewer acupoints (typically 1–3) not only facilitates analysis of the mechanism of therapeutic effects but also improve clinical practicality, enabling streamlined therapeutic designs. Moreover, distal acupoints may offer specific advantages for certain pain subtypes, which is worthy of further exploration. Factors such as needling techniques, modes of acupuncture stimulation, and adjunctive exercise modalities (e.g., active exercise, myofascial release, stretching) may also influence therapeutic efficacy (20). However, relevant analyses in the literature remain underexplored.

To address these research gaps, this study focused on acupoint selection strategies for AMP and selected RCTs employing 1–3 acupoints as the core dataset for analysis. CiteSpace, developed by Professor Chaomei Chen via Java, is a powerful bibliometric and visualization tool (21). Through CiteSpace, research hotspots were identified through multidimensional bibliometric analyses, including keyword clustering, high-frequency term analysis, and burst detection, thereby clarifying the application trends of core acupoint combinations. Through frequency statistics, influential factor screening, and spatial visualization of acupoint distributions, the empirical rationale and theoretical logic behind “core acupoint protocols” are further uncovered. The ultimate goal is to provide clinicians with referable, scientifically grounded and efficient acupoint selection strategies tailored to various subtypes of AMP.

## 2 Methods

### 2.1 Data sources and search strategy

Literature data were retrieved from the China National Knowledge Infrastructure (CNKI), WanFang data, VIP Chinese Science and Technology Periodical Database (VIP), SinoMed, Web of Science (WOS), PubMed, Embase, and Cochrane, from their inception to October 15, 2025. A combination of topic and free word was used. Detailed search strategies are presented in Supplementary material 1.

### 2.2 Inclusion and exclusion criteria

To ensure the accuracy and relevance, rigorous eligibility criteria were established. The inclusion criterion encompassed RCTs, observational research, and case reports. The exclusion criteria were as follows: (1) non-original studies, such as conference abstracts, dissertations, announcements, reviews, systematic reviews, or meta-analyses; (2) studies with those focused on chronic pain, identified by terms such as “chronic,” “refractory,” “old injury,” “old strain,” or “degenerative changes,” or those in which the primary symptoms were non-pain-related manifestations (e.g., numbness, weakness); (3) studies with musculoskeletal pain secondary to postoperative or visceral acute pain; (4) studies with incomplete information, such as missing full text, unclear needling protocols, or the use of proprietary treatment formulas; (5) studies in which more than three acupoints were used during the intervention; (6) interventions in which acupuncture was not the primary treatment modality, with dominant therapies, including herbal medicine, Tuina, or physical therapy; (7) secondary musculoskeletal disorders, such as stroke, Parkinson’s disease, etc.

### 2.3 Data processing and analysis

All included Chinese studies were exported in RefWorks format, and English studies in txt formats, and then secondary screened using NoteExpress 3.9.0.9588. The exported documents are saved as “download.txt” and imported into the visualization analysis tool CiteSpace 6.1.R6 to carry out scientific knowledge graph construction and visualization analysis of the keywords. After the database was constructed, all included studies were read in full, and core intervention information was extracted exclusively from the acupuncture treatment groups. For studies with population subgroups or intervention groups that all met the inclusion criteria, each group was treated as a separate analytical unit.

To ensure standardized and consistent data extraction, the following criteria were applied: (1) Modern medical diagnosis. (2) Pain location classification, based on the description in the studies, categorized into neck, shoulder, waist, hip-buttock, thigh, calf, knee, ankle, foot, elbow, wrist and hand. (3) Acupoint selection strategy, classified according to proximal acupoint selection and distal acupoint selection. Distal acupoint selection is defined as involving acupoints separated from the pain site by two or more joints (e.g., wrist, elbow, shoulder, hip-buttock, knee, ankle), while proximal acupoint selection involved fewer than two joints. The division of distal and proximal acupoints selection strategy in this paper is an operational definition employed specifically to facilitate cross-study comparisons, and does not represent an established consensus standard. Cases involving both were included in the analysis. (4) Needle angle, classified as “perpendicular needle insertion,” “oblique needle insertion” and “subcutaneous needle insertion.” (5) Whether exercise is involved, based on whether the study clearly indicates active or passive exercise, training, stretching, or other interventions during acupuncture, divided into “with exercise” and “without exercise.” (6) Type of acupuncture, classified as “body acupuncture,” “scalp acupuncture,” “auricular acupuncture,” “wrist-ankle acupuncture,” “hand acupuncture,” “foot acupuncture,” “eye acupuncture.” (7) Number of acupoints, recording the actual number of acupoints used during treatment, bilateral acupoints of the same name are counted as 1 acupoint. (8) Combined therapies, such as combined massage, TDP irradiation, hot compress, etc., are also recorded. (9) Specific acupoint, based on the *WHO International Standard Terminologies On Traditional Chinese Meidicine* and *National Standards of the People’s Republic of China* GB/T 12346-2021 (Meridian Acupoints), GB/T 13734-2008 (Auricular Acupuncture), GB/T 40997-2021 (Extra Acupuncture), GB/T 40997-2021 (Nomenclature and location of extra points in common), GB/T 21709.19-2009 (Wrist-Ankle Acupuncture), GB/T 21709.15-2021 (Ophthalmic Acupuncture), and GB/T 21709.2-2021 (Scalp Acupuncture) (22). For non-standard acupoint naming, the above-mentioned acupoints are matched based on the description of acupoint location. Auricular acupoints are represented as AP-X, wrist and ankle acupoints as WAA-X, ophthalmic acupoints as OA-X, and scalp acupoints as SA-X. Acupoints that are not found are described according to the EX-original description name and listed in Supplementary material 2. Proximal “tender points,” “pain points,” “sensitive points,” etc. are uniformly classified as “Ashi points.” Descriptions of distal Ashi points or sensitive points are uniformly classified as “EX-Ashi points.” If multiple eligible therapeutic strategies are reported in the same study, each strategy shall be included as an independent entry, and a metrological analysis shall be subsequently performed on all included entries. The protocol for this review was prospectively registered on the International Prospective Register of Systematic Reviews (PROSPERO) platform (Registration number: CRD420251155446).

### 2.4 Data analysis methods

**FIGURE 1:**
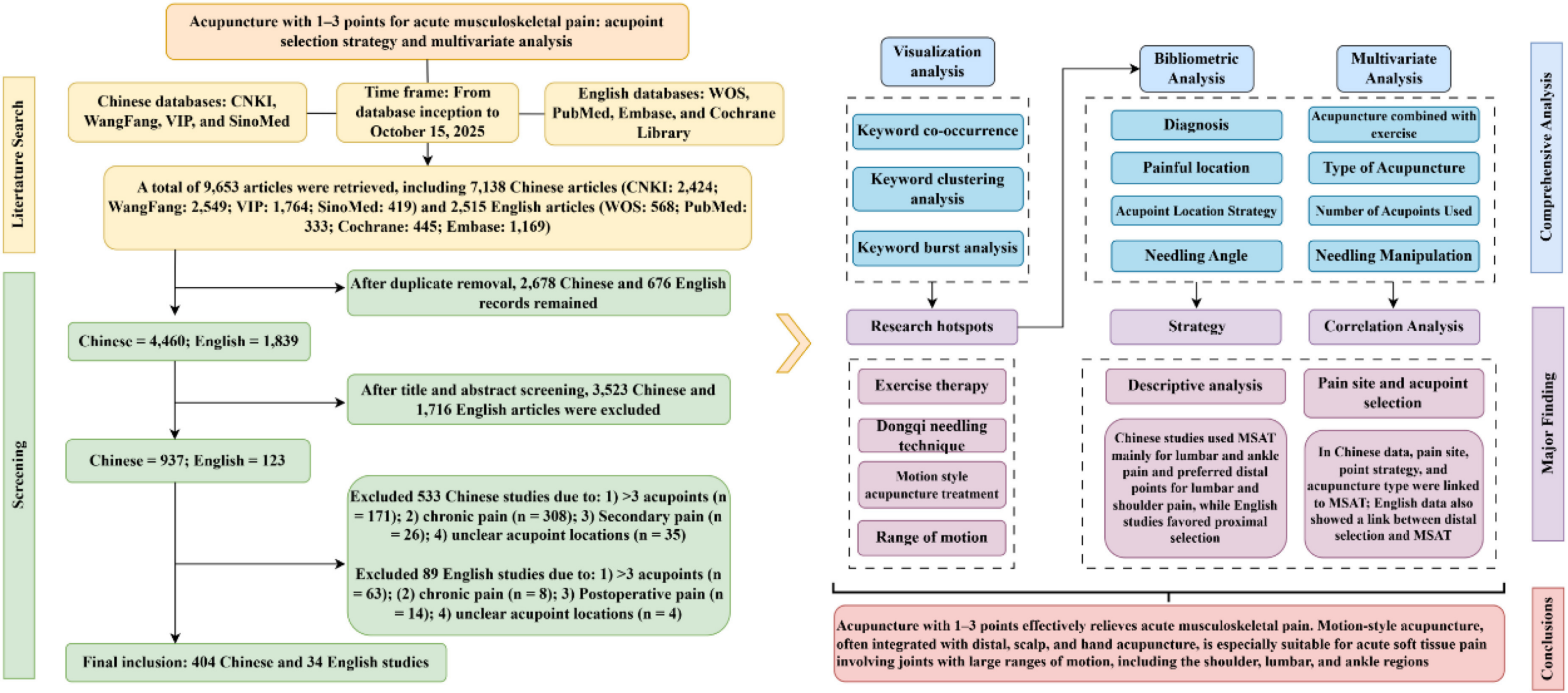
Inclusion criteria and literature screening process of this study.

First, the number of publications in both Chinese and English was counted to reveal the developmental trend in this research domain. To identify research hotspots and their evolutionary patterns, keyword clustering and burst detection were performed via CiteSpace version 6.1.R6. High-correlation research topics were extracted by adjusting the node connection strength (Degree). Secondly, Microsoft Excel was used to perform frequency statistics on the standardized data, and R 4.3.0 software was used to draw Sankey diagrams to statistically analyze the treatment pathways for pain in different parts of the body. “Whether combined with exercise” was used as the dependent variable, and other extracted variables were used as independent variables. Univariate binary logistic regression analysis was first performed to screen out variables with statistical significance (*p* < 0.05), and then they were included in a multivariate logistic regression model to further explore their independent association with the exercise combination factor. Based on the screened factors, acupoint selection strategies for specific locations were explored, and a spatial distribution graph of acupoints was generated. Through the above analysis, the potential patterns of acupoint selection strategies in the acupuncture treatment of AMP were explored, providing a reference for developing concise and effective clinical intervention protocols (Figure 1)

## 3 Results

### 3.1 Literature retrieval results

**FIGURE 2:**
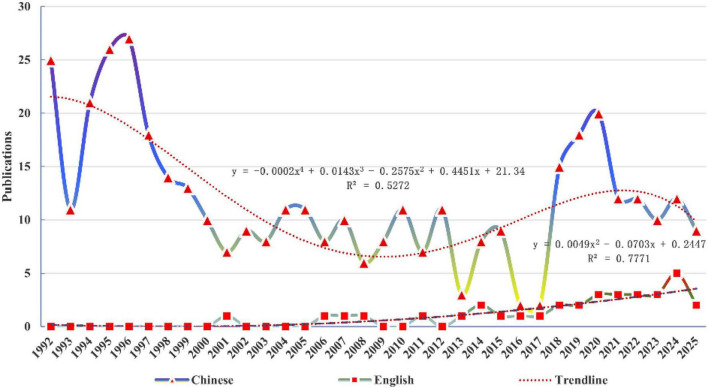
The number of publications each year from 1992 to 2025.

This study retrieved 2424 studies from CNKI, 2549 from Wanfang, 1764 from VIP, 419 from SinoMed, 568 from WOS, 333 from PubMed, 445 from Cochrane, 1169 from Embase. After applying inclusion and exclusion criteria, 404 Chinese and 34 English studies were included. The annual publication trends of Chinese studies exhibited a fluctuating evolutionary trajectory as “high-low-high”, which can be divided into three phases: an early high-productivity phase (1992–1998), a low-level fluctuation phase (1999-2017), and a subsequent resurgence (2018-present). The number of English studies was almost non-existent or extremely low in the early years but has shown an upward trend since 2010 (Figure 2).

### 3.2 CiteSpace keywords clustering analysis

**FIGURE 3:**
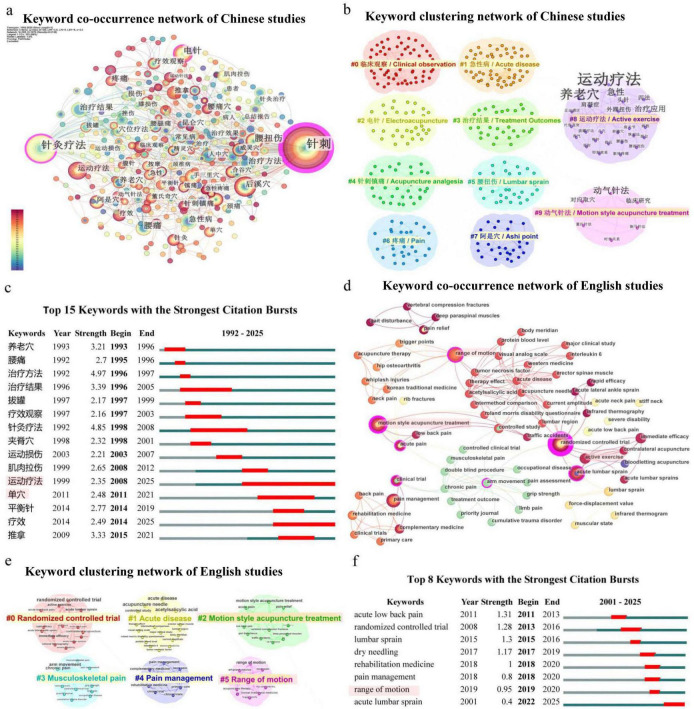
Keyword co-occurrence, clustering, and burst detection analysis in Chinese and English studies based on CiteSpace.

This study used CiteSpace 6.1.R6 to perform co-occurrence, clustering, and burst detection analysis on keywords in Chinese and English studies. The LLR algorithm was used to identify keywords from cited studies for annotation and clustering analysis to reveal research hotspots and evolutionary trends in the field of acupuncture treatment for AMP. Nodes represent keywords, and the size of the circles represents the frequency of keyword citation. Figures 3a,b show the keyword co-occurrence and clustering network of Chinese studies, forming a total of 10 clustering modules. The clustering modularity (Q) is 0.69, and the average silhouette coefficient (S) is 0.8608, indicating that the clustering structure has good significance and internal consistency. The identified clusters included “#0 Clinical observation,” “#1 Acute disease,” “#2 Electroacupuncture,” “#3 Treatment Outcomes,” “#4 Acupuncture analgesia,” “#5 Lumbar sprain,” “#6 Pain,” “#7 Ashi point,” “#8 Active exercise,” “#9 Dongqi needle technique.” Keyword timeline plots and burst detection analysis further reveal the dynamic evolution of research hotspots at different stages. Figure 3c reveal that early Chinese studies mainly focus on the selection of acupoints and therapeutic effects. After 2008, motion style acupuncture treatment, single acupoints, and balance acupuncture became prominent hotspots, gradually becoming a research focus in AMP. Figures 3d,e show the co-occurrence and clustering of keywords in English studies, identify 6 clustering modules with a clustering modularity(Q) is 0.8181 and the average silhouette coefficient(S) is 0.951, indicating high clustering quality. The results of keywords clustering included “#1 Randomized controlled trial,” “#2 Acute disease,” “#3 MSAT,” “#4 Pain management,” “#5 Range of motion” and etc., reflecting current research focus on clinical research of acute disease, pain management, joint mobility, and individualized treatment. The burst detection analysis showed that recent reaches concentrated on acupuncture intervention and pain management of lumbar sprain and low back pain (see Figures 3c,f for details). Overall, Chinese and English clinical research showed a surge research related to MSAT.

### 3.3 Literature extraction analysis

#### 3.3.1 Descriptive analysis

**FIGURE 4:**
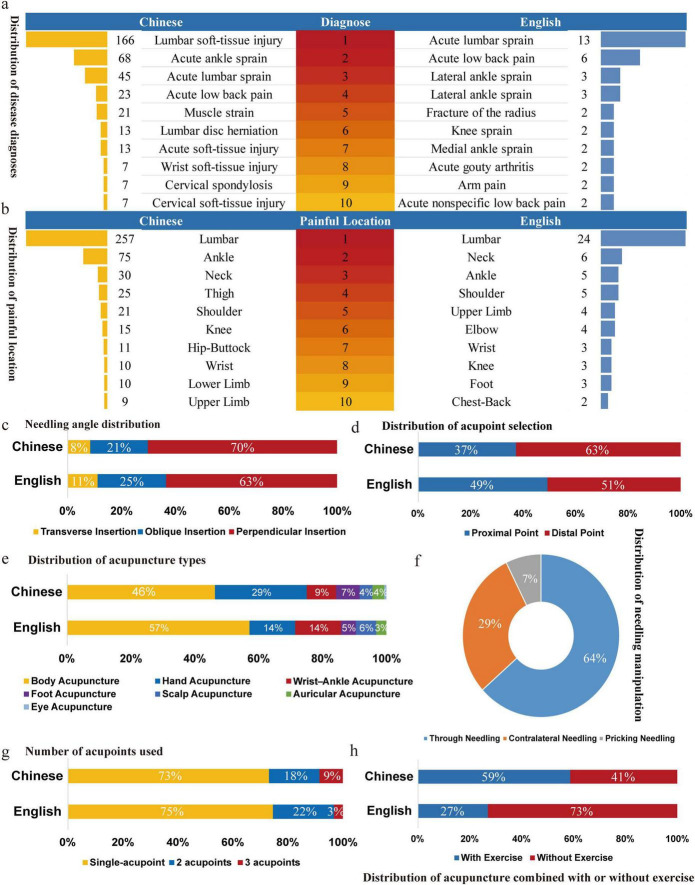
Descriptive analysis of core elements.

A total of 515 Chinese and 63 English therapeutic strategies were reviewed. In terms of disease spectrum, lumbar soft tissue pain is the most common disease across both language groups, with similar indications reported in each (Figure 4a). Pain localization analysis revealed a predominant focus on lumbar and ankle regions (Figure 4b). Perpendicular needle insertion was the most common needling angle (Figure 4c). In both Chinese and English studies, distal acupoints were selected more frequently than proximal ones (Figure 4d). Regarding acupuncture types, body acupuncture was the prevailing technique, but hand acupuncture predominated in Chinese studies, while English studies used wrist-ankle acupuncture more often (Figure 4e). Acupuncture methods were mainly described in Chinese studies, with Point-toward-point needle insertion being the most used method (Figure 4f). In terms of the number of acupoints, both Chinese and English studies prefer selecting single acupoint (Figure 4g). In a comparison of acupoint selection strategies and the distribution of acupuncture combined with exercise, Chinese research often incorporated exercise therapy, whereas English studies rarely combined acupuncture with exercise (Figure 4g).

#### 3.3.2 Pain location—trunk

**FIGURE 5:**
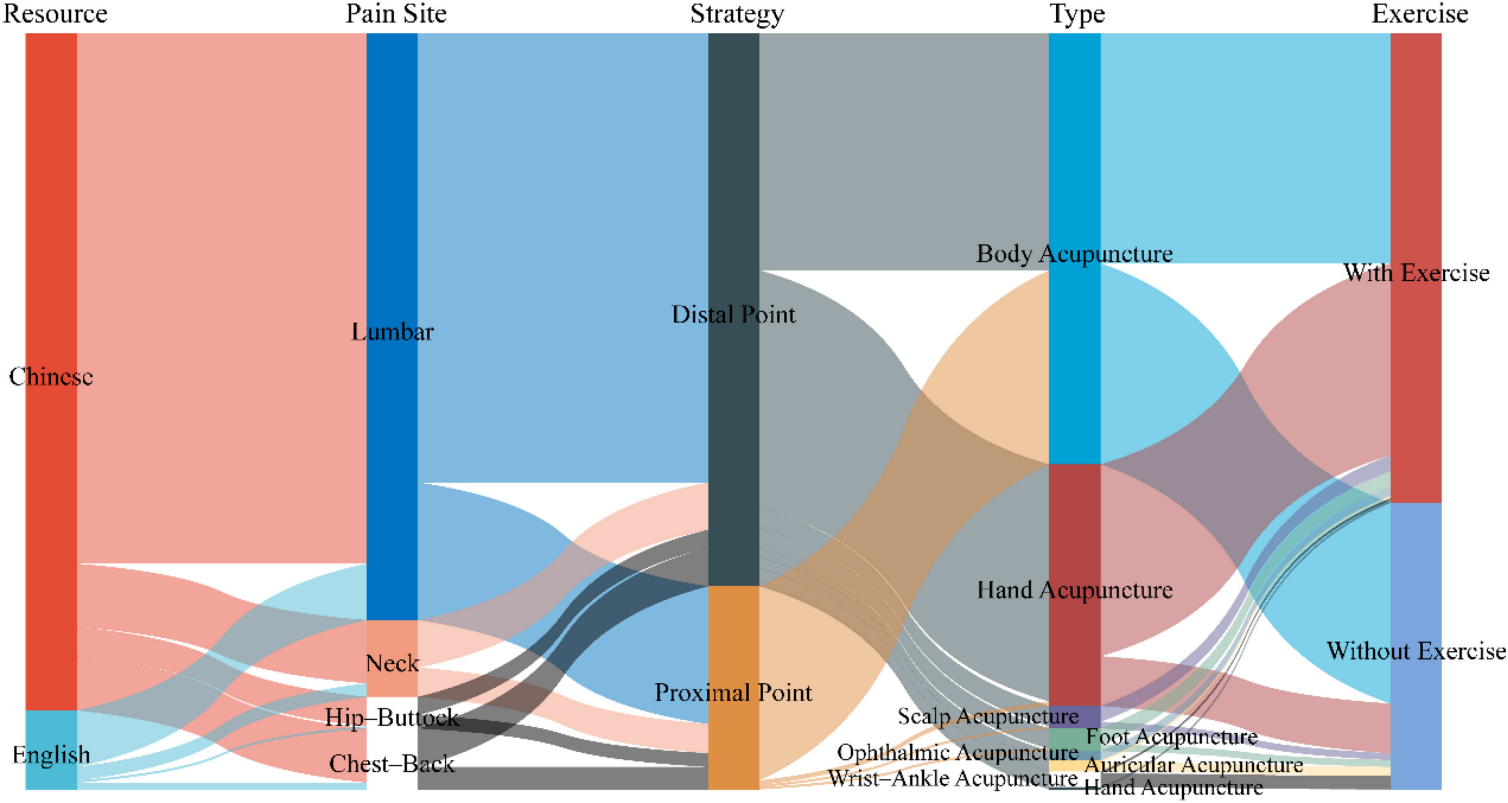
Sankey diagram of acupoint selection strategies for trunk pain.

Regarding acupoint selection for neck pain, Chinese studies often use SI3, SI6, and Ashi point in conjunction with exercise regimens, and Ashi point, EX-Jingtong, and EX-Zhongping in non-exercise regimens. English studies, however, often use LI10, LI16, and SI15 in conjunction with exercise regimens, and Ashi point and SI3 in non-exercise regimens. For chest and back pain, proximal acupoint selection is frequently used. Chinese studies use Ashi point, PC6, and SJ6 in conjunction with exercise regimens, and Ashi point, GB34, and EX-B2 in non-exercise regimens. English studies use Ashi point, CO14, and PC6 in non-exercise regimens. The lumbar region is the most frequently treated area in this study. Distal acupoint selection is frequently used for lumbar pain. Chinese studies use EX-UE7, SI3, and Ashi point in conjunction with exercise regimens, and Ashi point, EX-B2, and SI3 in non-exercise regimens. English studies use EX-UE7, LI11, and LR2 in conjunction with exercise regimens, and BL57, BL62, and EX-LE7 in non-exercise regimens. Regarding the selection of acupoints for hip-buttock pain, Chinese studies used Ashi point, BL65, and GB41 in combination with exercise regimens, and AP-AH7, Ashi point, and TE5 in non-combined exercise regimens. English studies used Ashi point in combination with exercise programs (see Figure 5 and Table 1 for details and see Supplementary material 2 for acupoint locations).

**TABLE 1:** Core acupoints for treating acute trunk musculoskeletal pain.

Resource	Pain site	With exercise	Without exercise
Chinese	Neck	SI3 (2); SI6 (2); Ashi point; EX-Chongzi; EX-Jingtong; GB39; LU8; SA-Ashi point; TE2; TE3; TE5	Ashi point (4); EX-Jingtong (3); EX-Zhongping (2); BL10; EX-Jingjiaji; GB39; LI18
	Chest–Back	Ashi point (4); PC6 (2); SJ6; LU10; BL60; BL10; ST16	Ashi point (5); GB34 (2); EX-B2 (2); PC6; PC7; HT7; LR3
	Lumbar	EX-UE7 (32); SI3 (24); Ashi point (10); GV26 (9); LI10 (9); SI6 (9); EX-Niushang (6); BL10 (5); BL2 (4); BL40 (3); GV28 (3); LI4 (3); OA-BL1 (3); BL59 (2); BL60 (2); EX-Ashi point (2); EX-B7 (2); TE5 (2); BL52; BL57; BL58; BL63; BL65; EX-B8; EX-Chixia; EX-Cuoshan; EX-Ezhong; EX-Face Yao; EX-HN9; EX-Linggu; EX-Tongling; EX-UE9; EX-Yaotong; GB39; GV14; GV24+; GV4; LI11; LI2; LI9; LR2; LR3; OA-2; SA-Ashi point; SA-GV20; SA-Renzifengjian; SI4; SP6; ST38; WAA-Down 5	Ashi point (20); EX-B2 (6); SI3 (6); GV26 (4); WAA-Down 5 (3); EX-UE7 (3); BL23 (2); BL40 (2); LU5 (2); TE6 (2); WAA-Down 6 (2); EX-Ashi point (5); EX-Yaotu (2); AP-AH9; BL25; BL63; EX-Daimaihou; EX-Tunzhong; EX-Xiashandian; EX-Yaotong; GB20; GV2; GV3; KI4; SA-GB15; SI6; ST40
	Hip–Buttock	Ashi point; BL65; GB41; LI10; LU10; SA-Ashi point; SA-GV20	AP-AH7; Ashi point (4); TE5
English	Neck	LI10; LI16; SI15; SI3; TE15; TE3	Ashi point (2); SI3
	Chest–Back	–	Ashi point; CO14; PC6; TF4
	Lumbar	EX-UE7 (2); LI11 (2); LR2 (2); EX-B2; AH9; GV16; GV24 + ; SA-GV16; SA-BL2; SI3	Ashi point (5); SI3 (3); EX-B2; BL40; BL57; BL62; EX-LE7; GV26; SA-BL2; SI6; ST36; WAA-Down 5; WAA-Down 6
	Hip–Buttock	–	Ashi point

#### 3.3.3 Pain location—upper limb

**FIGURE 6:**
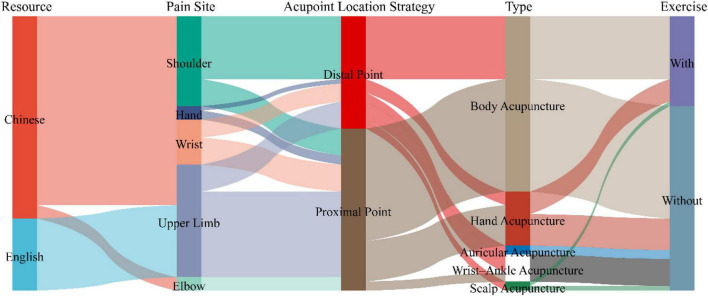
Sankey diagram of acupoint selection strategies for upper limb pain.

Hand pain treatment often prioritizes proximal acupoint selection. Chinese studies using exercise regimens selected SI3, SI6, and Ashi points, while non-exercise regimens selected Ashi points, SP6 Ashi points, and TE5. English studies using non-exercise regimens selected Ashi points, PC6, and WAA-Up 5. For wrist pain, proximal acupoints are often selected. Chinese studies using exercise regimens include Ashi point, GB29, and SA-Yidian, while non-exercise programs use Ashi point, AP-SF2, and EX-Ashi point. English studies with non-exercise regimens use Ashi point, PC6, and WAA-Up 5. For elbow pain, proximal acupoints are often selected. Chinese studies with non-exercise programs use AP-SF3, Ashi point, and WAA-Ashi point. Similarly, English studies with non-exercise programs use Ashi point, LI10, LI11, and WAA-Up 5. The acupoint selection strategy for shoulder pain often focuses on distal acupoints, combining body acupuncture with exercise therapy. Chinese studies use SI3, SI6, and Ashi point in conjunction with exercise regimens, and Ashi point, EX-Zhongping, and AP-SF4,5 in non-compliance exercise regimens. English studies use Ashi point, EX-Zhongping, and LI14 in conjunction with exercise regimens (Figure 6 and Table 2). Acupoint locations are shown in Supplementary material 2.

**TABLE 2:** Core acupoints for treating acute upper limb musculoskeletal pain.

Resource	Pain site	With exercise	Without exercise
Chinese	Hand	Ashi point; SP6	Ashi point; TE5
	Wrist	Ashi point (4); GB29; SA-Yidian; SP6	Ashi point (2); AP-SF2; EX-Ashi point; WAA-Ashi point
	Elbow	–	AP-SF3; Ashi point (2); WAA-Ashi point
	Shoulder	ST38 (3); LI15 (2); Ashi point; EX-Zhongping; SI3; SI6; ST31	Ashi point (2); EX-Zhongping (3); AP-SF4,5; EX-Jinggen; TE5; WAA-Ashi point
	Upper Limb	Ashi point	Ashi point (5); LI4 (2); TE5
English	Wrist	–	Ashi point; PC6; WAA-Up 5
	Elbow	Ashi point	Ashi point; LI10; LI11; WAA-Up 5
	Shoulder	–	Ashi point (3); EX-Zhongping; LI14; WAA-Up 4; WAA-Up 5
	Upper Limb	–	LI4 (2); Ashi point; LR3; SA-GV20

#### 3.3.4 Pain location—lower limb

**FIGURE 7:**
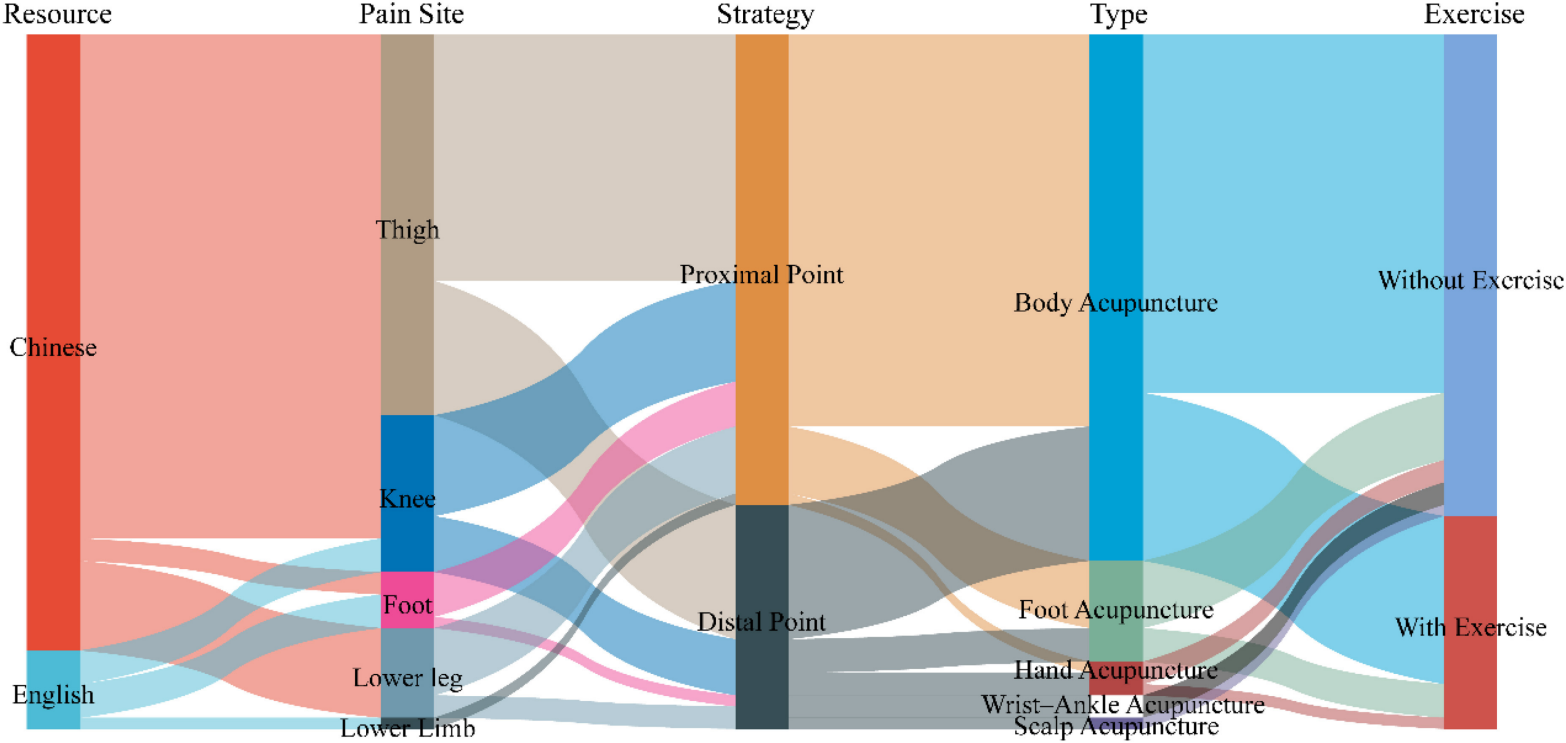
Sankey diagram of acupoint selection strategies for lower limb pain.

For thigh pain, proximal acupoints are often selected. Chinese studies using exercise regimens include Ashi point, BL57, and EX-Xinmen while non-exercise regimens include Ashi point, BL63, and EX-Jianqian. For knee pain, proximal acupoints are often selected. Chinese studies using exercise programs include Ashi point and TE10 while non-exercise regimens include Ashi point, LI11, and SP8. English studies using exercise programs include Ashi point while non-exercise regimens include Ashi point, WAA-Down 3, and WAA-Down 4. For ankle pain, distal acupoints are often selected. Chinese studies without exercise regimens include EX-Xiaojie, Ashi point, and TE4. Similarly, English studies using without exercise programs include Ashi point, TE5, and WAA-Ashi point. For foot pain, proximal acupoints are often selected. Chinese studies without exercise regimens include Ashi point and English studies without exercise programs include Ashi point, SP6, and ST36 (Figure 7 and Table 3). Acupoint locations are shown in Supplementary material 2.

**TABLE 3:** Core acupoints for treating acute lower limb musculoskeletal pain.

Resource	Pain site	With exercise	Without exercise
Chinese	Thigh	Ashi point (4); BL57 (1); EX-Xinmen (1); GB30 (1); GB34 (1); LI14 (1); LR3 (1); PC2 (1); SP3 (1); ST36 (1)	Ashi point (15); BL63 (1); EX-Jianqian (1); EX-UE3 (1); SA-MS7 (1); SP10 (1); ST36 (1)
	Knee	Ashi point (1); TE10 (1)	Ashi point (5); LI11 (1); SP8 (1); TE5 (1); WAA-Ashi point (1)
	Lower leg	Ashi point (2); ST43 (1)	Ashi point (3); BL58 (1); BL63 (1)
	Ankle	EX-Xiaojie (9); Ashi point (8); TE4 (5); PC7 (3); EX-Ashi point (3); KI3 (2); LI9 (2); LU9 (2); BL2 (1); BL60 (1); EX-UE7 (1); LI5 (1); SA-Xingdian (1); SI3 (1); SI4 (1); SI6 (1); ST36 (1); AP-HX6, 7i (1)	Ashi point (10); TE5 (2); WAA-Ashi point (2); AP-AH3 (1); EX-Fugudou (1); EX-Wuhu (1); GB34 (1); GB36 (1); GB39 (1); PC6 (1); ST41 (1)
	Foot	–	Ashi point (2)
English	Lower Limb	–	LR3 (1); ST34 (1); ST36 (1)
	Knee	Ashi point (1)	Ashi point (1); WAA-Down 3 (1); WAA-Down 4 (1)
	Ankle	GB40 (1); GB41 (1)	Ashi point (2); WAA-Down 2 (1); WAA-Down 5 (1)
	Foot	–	Ashi point (1); SP6 (1); ST36 (1)

#### 3.3.5 Correlation analysis between influencing factors and specific parts

**FIGURE 8:**
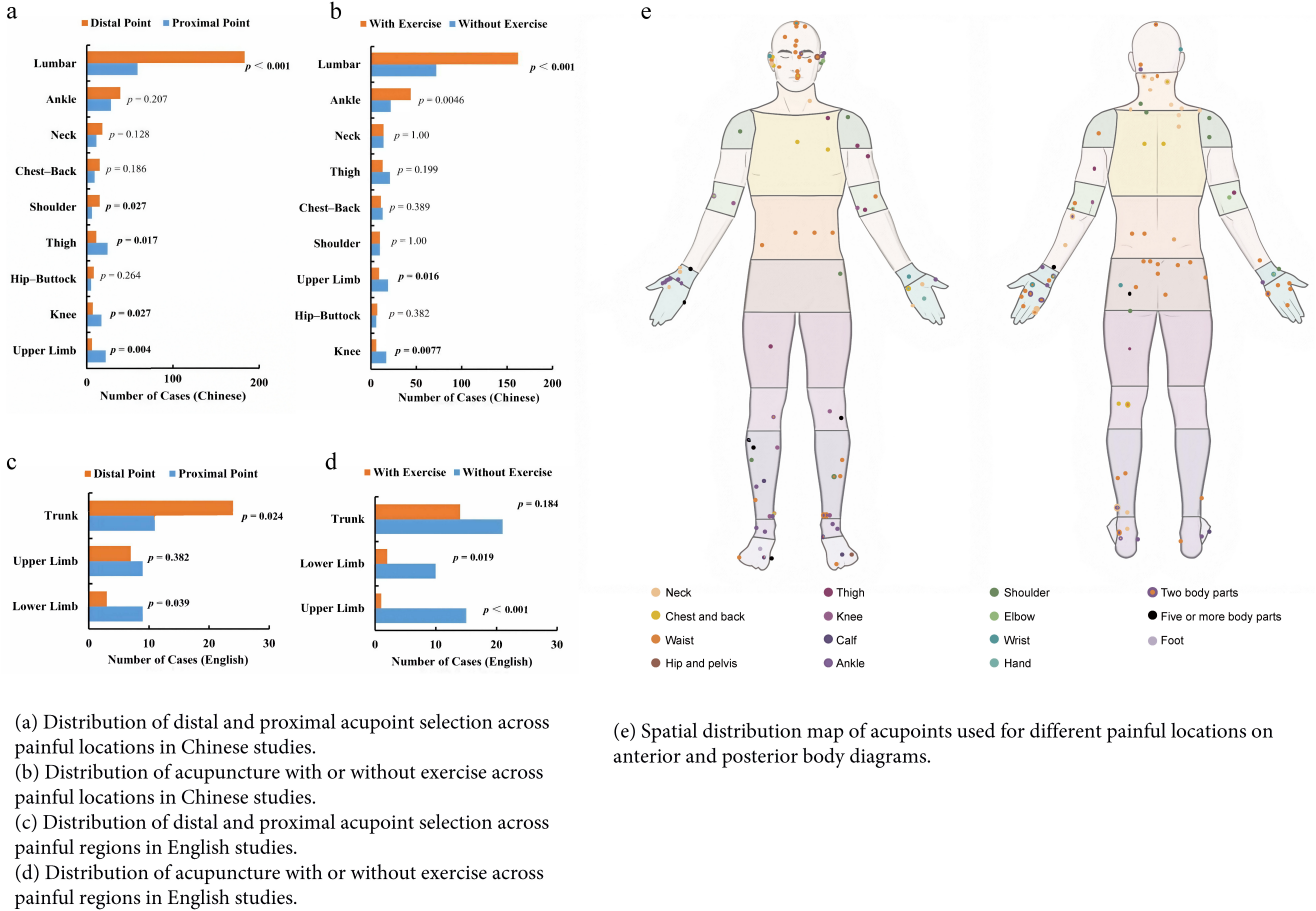
Correlation analysis between influencing factors and specific parts.

This section explores the selection of distal or proximal acupoints and combination of exercise for pain in different areas. When pain occurs in the waist, ankle, neck, chest-back, shoulder, and hip-buttock, distal acupoints are used more frequently than proximal acupoints, especially in the waist and shoulder. However, for pain in the thigh, knee, and upper limb, proximal acupoints are used significantly more frequently than distal aucpoints. Similarly, English studies support the preference for distal acupoints for trunk pain and proximal acupoints for limb pain (Figures 8a,c). The spatial distribution of acupoints also supports the distribution of locations and acupoints, as shown in Figure 8e. Similarly, in Chinese studies on the use of exercise for pain in different locations, acupuncture combined with exercise is more frequently used for pain in the waist and ankle, but less frequently for pain in the upper limb and knee. English studies use acupuncture combined with exercise less frequently, as shown in Figures 8b,d.

### 3.4 Correlation analysis

**FIGURE 9:**
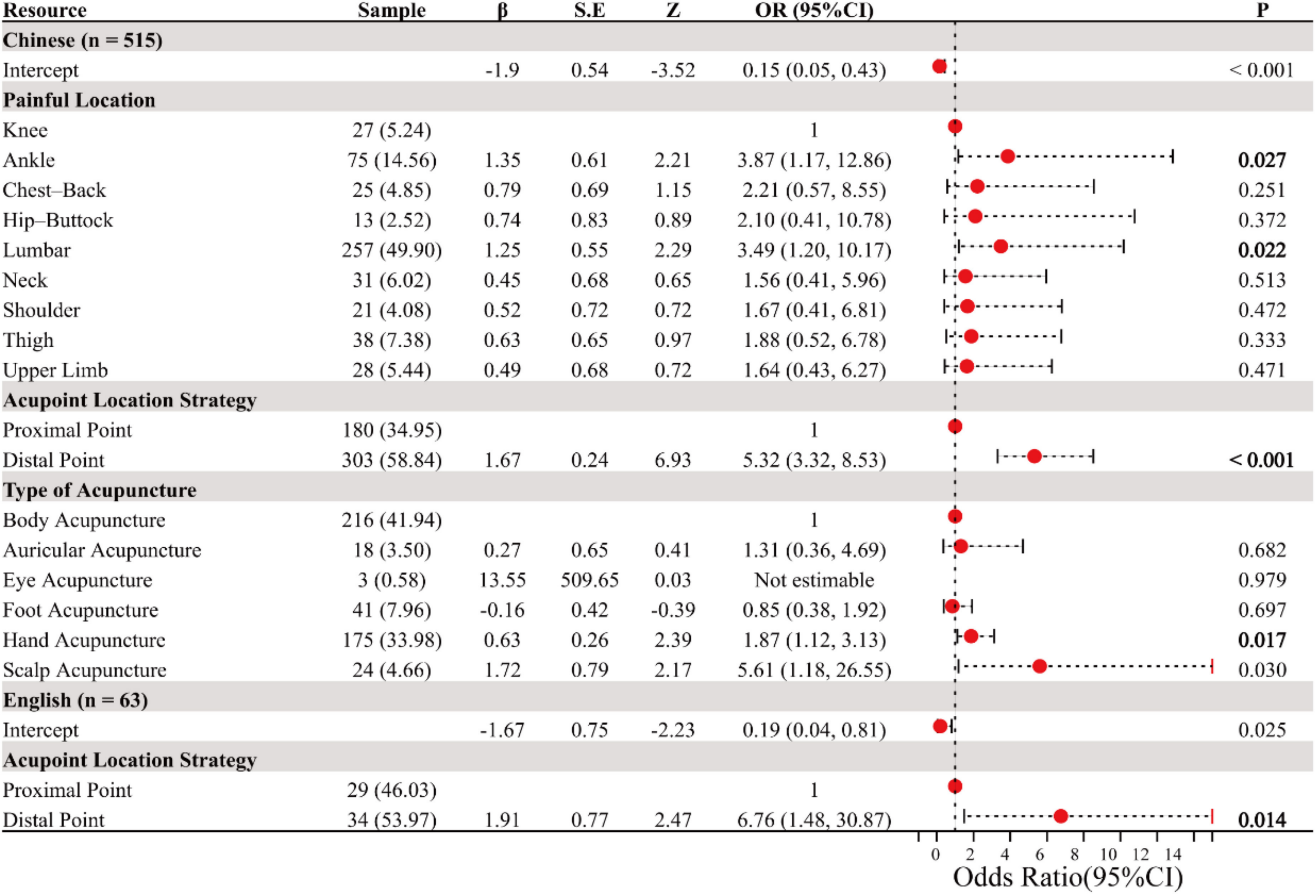
Multivariate correlation analysis.

Using “whether combined with exercise” as the dependent variable, and selecting “Painful Location,” “Acupoint Location Strategy,” “Needling Manipulation,” “Needling Angle,” “Type of Acupuncture,” and “Number of Acupoints” as independent variables, univariate and multivariate logistic regression analyses were conducted. In Chinese studies, univariate analysis revealed significant associations between factors such as “Painful Location,” “Acupoint Location Strategy,” and “Type of Acupuncture” with exercise compliance. Multivariate regression analysis further indicated that Ankle pain (OR = 3.87, 95% CI: 1.17–12.86, *p* = 0.027), Lumbar pain (OR = 3.49, 95% CI: 1.20–10.17, *p* = 0.022), Distal acupoint selection (OR = 5.32, 95% CI: 3.32–8.53, *p* < 0.001), Hand acupuncture (OR = 1.87, 95% CI: 1.12–3.13, *p* = 0.017), and Scalp acupuncture (OR = 5.61, 95% CI: 1.18–26.55) were significantly correlated with exercise. Although the sample size of English literature is small, a significant correlation was still observed between distal acupoint selection and combined exercise (OR = 6.76, 95% CI: 1.48–30.87, *p* = 0.014), as shown in Figure 9.

## 4 Discussion

This study underscored acupuncture’s potential significance in treating AMP. Acupoint sensitization appears to bridge disease diagnosis and treatment (23, 24). Under pathological conditions, sensitized acupoints exhibit a neurogenic inflammation, often resulting in the aggregation of mast cells and immune inflammatory cells in the skin and subcutaneous tissues, as well as the release of inflammatory mediators and active substances (25, 26). This is thought to form a “sensitization pool” or “inflammatory soup” locally or distally, manifesting as hypersensitivity (aches, swelling, pain, itching) or hyposensitivity in the local skin and muscles, changes in temperature and blood flow, or abnormal skin morphology (nodules, cords, pigmentation), etc. Zhu et al. summarized the patterns of acupoint sensitization in more than 20 types of visceral diseases in China, finding that sensitized acupoints are distributed throughout the body, with dynamic changes in acupoint distribution and reduced pain thresholds (27). Acupuncture targeting these acupoints can enhance acupoint function and potentially exert a significant effect with minimal stimulation, thus possibly improving clinical efficacy. Other studies have found that sensitized acupoints dynamically shift from a “resting state” to an “activated state,” with the number of sensitized acupoints decreasing as the disease improves (23, 28). Consequently, acupuncture targeting these points can yield more pronounced clinical outcomes. Core acupoints are might be viewed as a collection of sensitized acupoints, derived from extensive clinical experience. Distal acupoint selection is particularly unique, may exert its effects through somatosensory-autonomic reflex mechanisms (29–31). These reflexes are believed to begin with the activation of peripheral nerves derived from neurons in the dorsal root ganglion (DRG) and/or trigeminal ganglion, followed by the spinal cord transmitting sensory information to the brain, activating peripheral autonomic pathways, and ultimately regulating various physiological functions of the body.

Acupuncture combined with exercise therapy is considered an important method for treating AMP, often used in conjunction with hand acupuncture, scalp acupuncture, and distal acupoint selection. Acupuncture combined with exercise has emerged as an increasingly recongnized therapeutic modality. In Chinese, it is mainly described as “Dongqi needle technique” or “exercise therapy,” while English studies use the term MSAT. This approach integrates acupuncture with active or passive movement while needles are retained, potentially amplifying the stimulation effect by moving the muscles being needled, which may result in better regulation of central nervous system neurons and thus a better analgesic effect compared to traditional acupuncture (32, 33). As a rapidly developing integrated approach in recent years, MSAT has demonstrated superior analgesic and functional improvement effects in numerous clinical studies (34, 35). The core characteristics include: (1) Acupoints are usually located outside the affected area, especially on the distal healthy side, facilitating movement and avoiding increased local burden. (2) Active, passive, or guided movements are performed simultaneously during acupuncture, such as breathing exercises, Daoyin (guiding and stretching), and muscle stretching. (3) Acupoints are selected concisely, often focusing on the Five-Shu points, Yuan-source points, Xi-cleft points, and Dong’s extraordinary points (32). Some studies have also shown that applying Ashi points to the injured area and simultaneously inducing local functional activity can also achieve favorable results (36, 37). MSAT’s advantage appears to lie in its regulatory capacity to modulate the central nervous system. By retaining the needles and guiding the patient to perform active or passive movements, it is suggested to enhance the activation of subcutaneous high-density A and C fibers, inhibits the activity of long-term potentiation (LTP) and wide dynamic range (WDR) neurons in the spinal cord, and depresses the transmission of pain signals (38, 39). Moreover, acupuncture promotes the release of endogenous opioid peptides and various neurotransmitters (40, 41). Regarding acupoint selection strategies, a systematic review indicated that both distal and proximal acupoints exert comparable pain-relief effects through synergistic regulation of peripheral and central nervous systems (42). Distal acupoints can influence the limbic-paralimbic-neocortical network, producing a broad-based central analgesic effect (43). In this study, distal acupoint selection was used as a closely related method of MSAT, forming an intervention model of “sensitization modulation + distal activation” together with hand acupuncture and scalp acupuncture. For example, scalp acupuncture combined with rehabilitation exercises can improve gross motor function and daily living abilities in children with cerebral palsy (44). Hand acupuncture with active exercise can significantly relieve pain and movement disorders in patients with acute neck pain (45). Collectively, these results suggest that combining distal sensitized acupoints with motor stimulation may be an optimized treatment strategy (32, 46).

Compared with current consensus guidelines of acupuncture treatment for acute pain, this study aimed to provide a relatively objective and quantitative basis for acupoint selection and proposes, and explores the potential therapeutic advantages of MSAT. Currently, many studies advocate following the consensus on acupuncture intervention for acute pain in the emergency department (5, 47–49). This study, through the Delphi consensus process and following the guidance of the STRICTA framework, emphasized a single targeted treatment course. In most cases of acute pain, distal acupoints are used as primary acupoints, with local acupoints as secondary acupoints. The consensus also reflects various acupuncture methods, such as stimulating local trigger points to induce fasciculations, distal contralateral acupoint selection, and joint-corresponding acupoint selection. Our research results were based on bibliometric and data-driven analysis of clinical studies using a “1-3 acupoints” protocol to treat AMP. These results, along with the consensus, emphasized the principle of distal acupoint selection or anatomical correspondence acupoint selection. This study further explores the location of the disease and acupoint selection strategies. Logistic regression analysis underscored the synergistic value of combining distal, scalp, or hand acupoints with exercise-based therapy. These findings may complement existing consensus treatment approaches. This study provides new evidence that low-dose acupuncture combined with targeted neuromuscular activation may offer a potentially effective alternative in certain acute pain situations.

Current research still has certain limitations. (1) Because the specific modalities, intensity, frequency, and duration of the combined exercise interventions varied substantially across the included studies, we dichotomized exercise into a simple binary variable (with or without). Consequently, the inherent heterogeneity of these exercise regimens may compromise the stability, interpretability, and reproducibility of the odds ratio (OR) estimates. We acknowledge this oversimplification as a methodological limitation of the present study. (2) Although spatial acupoint patterns are evident in clinical practice, the absence of three-dimensional imaging or musculoskeletal structure restricts the mechanistic precision between “specific acupoints and specific pain locations.” (3) Given the significant volume disparity between Chinese and English literature, we did not intend a strict equivalent comparison; rather, to comprehensively identify acupoint patterns and hotspots, the English findings should be interpreted primarily as descriptive and exploratory. (4) Considering that this type of disease includes multiple diseases, there are no statistical outcome indicators, which has a certain impact on the credibility of the acupoint selection strategy. (5) The classification method for distal and proximal acupoints may still involve a certain degree of subjectivity, which could potentially affect the robustness and interpretability of the analytical results.

## 5 Conclusion

Treatment of AMP using a limited set of core acupoints is clinically feasible, with MSAT being the most representative technique, exhibiting a clear site-specific acupoint selection pattern. Multivariate analysis further reveals that this strategy is often used in conjunction with distal acupoint selection, hand acupuncture, and scalp acupuncture, showing particularly significant potential therapeutic advantages in acute soft tissue injuries of the lumbar and ankle regions.

## Data Availability

The original contributions presented in this study are included in the article/Supplementary material, further inquiries can be directed to the corresponding authors.

## References

[1] GBD 2016 Disease and Injury Incidence and Prevalence Collaborators. Global, regional, and national incidence, prevalence, and years lived with disability for 328 diseases and injuries for 195 countries, 1990–2016: a systematic analysis for the Global Burden of Disease Study 2016. *The Lancet.* (2017) 390:1211–59. 10.1016/S0140-6736(17)32154-2 28919117 PMC5605509

[2] Hsu JR Mir H Wally MK Seymour RB. Clinical practice guidelines for pain management in acute musculoskeletal injury. *J Orthop Trauma*. (2019) 33:e158–82. 10.1097/BOT.0000000000001430 30681429 PMC6485308

[3] Fontanez R Guasp WR Ramirez H de Jesús K Conde JC González J et al.. Musculoskeletal conditions in the emergency room: a teaching opportunity for medical students and residents. *Puerto Rico Health Sci J.* (2021) 40:68. PMC9119411 34543564

[4] Baker B Kessler K Kaiser B Waller R Ingle M Brambilla S et al.. Non-traumatic musculoskeletal pain in Western Australian hospital emergency departments: a clinical audit of the prevalence, management practices and evidence-to-practice gaps. *Emerg Med Australas*. (2019) 31:1037–44. 10.1111/1742-6723.13305 31090200

[5] Eucker SA Glass O Knisely MR O’Regan A Gordee A Li C et al.. An adaptive pragmatic randomized controlled trial of emergency department acupuncture for acute musculoskeletal pain management. *Ann Emerg Med.* (2024) 84:337–50. 10.1016/j.annemergmed.2024.03.014 38795078

[6] de Sire A Marotta N Lippi L Scaturro D Farì G Liccardi A et al.. Pharmacological treatment for acute traumatic musculoskeletal pain in athletes. *Medicina*. (2021) 57:1208. 10.3390/medicina57111208 34833426 PMC8618079

[7] Yunshan L Chengli X Peiming Z Haocheng Q Xudong L Liming L. Integrative research on the mechanisms of acupuncture mechanics and interdisciplinary innovation. *BioMed Eng OnLine.* (2025) 24:30. 40055719 10.1186/s12938-025-01357-w PMC11889876

[8] Gao S Yi Z Liao F.. *Ling Shu Quan Yong.* Jinan: Jinan Publishing House (2007).

[9] Vas J Ortega C Olmo V Hernandez L Medina I Seminario JM et al.. Single-point acupuncture and physiotherapy for the treatment of painful shoulder: a multicentre randomized controlled trial. *Rheumatology.* (2008) 47:887–93. 10.1093/rheumatology/ken040 18403402

[10] Tiwari S Sapkota N. Is single-point acupuncture effective in treating acute low back pain? *Clin Case Rep.* (2021) 9:e05130. 10.1002/ccr3.5130 34853688 PMC8614094

[11] Li T Litscher G Zhou Y Song Y Shu Q Chen L et al.. Effects of acupuncture and moxibustion on heart rate variability in chronic fatigue syndrome patients: regulating the autonomic nervous system in a clinical randomized controlled trial. *Complement Ther Med*. (2025) 92:103184. 10.1016/j.ctim.2025.103184 40315935

[12] Seaman LA LaVergne D Walden AL Watson DP Yurasek FA Hudson M et al.. Clinician views on acupuncture for acute pain care in a busy urban emergency department. *Complement Therapies Clin Pract.* (2025) 59:101982. 10.1016/j.ctcp.2025.101982 40199183

[13] Tupetz A Frazier M O’Regan A Knisely M TumSuden O Walker E et al.. Participant experiences receiving acupuncture for acute musculoskeletal pain in an emergency department: a qualitative evaluation. *PLoS One*. (2025) 20:e0318345. 10.1371/journal.pone.0318345 39937807 PMC11819596

[14] Han Q Wang F. Electroacupuncture at GB20 improves cognitive ability and synaptic plasticity via the CaM–CaMKII–CREB signaling pathway following cerebral ischemia–reperfusion injury in rats. *Acupunct Med.* (2024) 42:23–31. 10.1177/09645284231202805 38126262

[15] Zhang Y Lu H Ren X Zhang J Wang Y Zhang C et al.. Immediate and long-term brain activation of acupuncture on ischemic stroke patients: an ALE meta-analysis of fMRI studies. *Front Neurosci*. (2024) 18:1392002. 10.3389/fnins.2024.1392002 39099634 PMC11294246

[16] Liu R Moe AAK Liu W Zoghi M Jaberzadeh S. Does acupuncture at motor-related acupoints affect corticospinal excitability? A systematic review and meta-analysis. *J Integr Med*. (2025) 23:113–25. 10.1016/j.joim.2025.02.004 40097324

[17] Chen P Jin X Yu D Chen X Lin Y Wu F et al.. Efficacy of acupuncture on lower limb motor dysfunction following stroke: a systematic review and meta-analysis of randomized controlled trials. *PLoS One*. (2025) 20:e0312918. 10.1371/journal.pone.0312918 40333934 PMC12057971

[18] Qu J Du Y Jing J Wang J Bu L Wang Y. Short-term longitudinal study on brain network informatics of stroke patients under acupuncture and motor imagery intervention. *IEEE J Biomed Health Inform*. (2025) 29:3356–65. 10.1109/JBHI.2025.3527074 40031051

[19] Kim YJ Ku J Cho S Kim HJ Cho YK Lim T et al.. Facilitation of corticospinal excitability by virtual reality exercise following anodal transcranial direct current stimulation in healthy volunteers and subacute stroke subjects. *J Neuroeng Rehabil.* (2014) 11:124. 10.1186/1743-0003-11-124 25135003 PMC4148539

[20] Wie HS Kim SN. Therapeutic components of acupuncture stimulation based on characteristics of sensory nerve and nervous signaling pathway. *J Integr Med*. (2025) 23:106–12. 10.1016/j.joim.2025.02.002 40069035

[21] Chen C. Searching for intellectual turning points: progressive knowledge domain visualization. *Proc Natl Acad Sci.* (2004) 101(Suppl_1):5303–10. 10.1073/pnas.0307513100 14724295 PMC387312

[22] Lim S. WHO standard acupuncture point locations. *Evid Based Complement Alternat Med*. (2010) 7:167–8. 10.1093/ecam/nep006 19204011 PMC2862941

[23] Li R Liu M Lv Y Jing F. Mast cells in acupoint sensitization: mechanisms and Research Advances. *Am J Chin Med*. (2025) 53:1265–84. 10.1142/S0192415X25500491 40626405

[24] Gao X-Y. Acupoint sensitization: bond of disease diagnosis and therapy. *Zhen ci yan jiu= Acupunct Res.* (2025) 50:504–12. 10.13702/j.1000-0607.20250250 40390607

[25] Cui X Liu K Gao X Zhu B. Advancing the understanding of acupoint sensitization and plasticity through cutaneous C-Nociceptors. *Front Neurosci*. (2022) 16:822436. 10.3389/fnins.2022.822436 35620665 PMC9127573

[26] Cui X Zhang Z Xi H Liu K Zhu B Gao X. Sympathetic-sensory coupling as a potential mechanism for acupoints sensitization. *J Pain Res.* (2023) 16:2997–3004. 10.2147/JPR.S424841 37667684 PMC10475306

[27] Xu WJ Cui X Liu K Zhu B Gao XY. [Roles of nociceptors in acupoint sensitization: recent advances]. *Zhen Ci Yan Jiu*. (2021) 46:1048–56. 10.13702/j.1000-0607.20210313 34970883

[28] Tan H Tumilty S Chapple C Liu L Othman R Baxter GD. Acupoints sensitization in people with and without chronic low back pain: a matched-sample cross-sectional study. *J Back Musculoskelet Rehabil.* (2023) 36:137–46. 10.3233/BMR-210297 35871318

[29] Liu S Wang Z Su Y Qi L Yang W Fu M et al.. A neuroanatomical basis for electroacupuncture to drive the vagal-adrenal axis. *Nature*. (2021) 598 641–5. 10.1038/s41586-021-04001-4 34646018 PMC9178665

[30] Ma Q. Somatotopic organization of autonomic reflexes by acupuncture. *Curr Opin Neurobiol.* (2022) 76:102602. 10.1016/j.conb.2022.102602 35780689

[31] Hsieh YL Hong CZ Liu SY Chou LW Yang CC. Acupuncture at distant myofascial trigger spots enhances endogenous opioids in rabbits: a possible mechanism for managing myofascial pain. *Acupunct Med*. (2016) 34:302–9. 10.1136/acupmed-2015-011026 27143259

[32] Chen Y Zhu F Zhu Y Duan Y Bai Z. The applications and prescriptions of motion style acupuncture treatment for musculoskeletal pain: a scoping review of clinical controlled trials. *J Pain Res.* (2025) 18:3275–87. 10.2147/JPR.S529676 40613071 PMC12223269

[33] Fu Y Xu Y Wu HX Wang SS. [Dong’s extraordinary point needling technique combined with medication for postoperative complications of anal fistula: a randomized controlled trial]. *Zhongguo Zhen Jiu*. (2023) 43:916–20. 10.13703/j.0255-2930.20220911-k0003 37577888

[34] Jeon J-H Woo H-J Ha W-B Geum JH Park SH Lee JH et al.. Domestic clinical research trends of motion-style acupuncture treatment: a scoping review. *J Korean Med Rehabil.* (2022) 32:19–32. 10.18325/jkmr.2022.32.4.19

[35] Kim DY Ha IH Kim JY. Graded exercise with motion style acupuncture therapy for a patient with failed back surgery syndrome and major depressive disorder: a case report and literature review. *Front Med*. (2024) 11:1376680. 10.3389/fmed.2024.1376680 38651058 PMC11034520

[36] Kim D Lee YJ Ha I-H. A scoping review of clinical research on motion style acupuncture treatment. *Perspect Integr Med.* (2023) 2:65–76. 10.56986/pim.2023.06.001

[37] Kim D Park KS Lee JH Ryu WH Moon H Park J et al.. Intensive Motion Style Acupuncture Treatment (MSAT) is effective for patients with acute whiplash injury: a randomized controlled trial. *J Clin Med*. (2020) 9:2079. 10.3390/jcm9072079 32630663 PMC7408694

[38] Fan Z Dou B Wang J Wu Y Du S Li J et al.. Effects and mechanisms of acupuncture analgesia mediated by afferent nerves in acupoint microenvironments. *Front Neurosci.* (2024) 17:1239839. 10.3389/fnins.2023.1239839 38384495 PMC10879281

[39] Chen T Zhang WW Chu YX Wang YQ. Acupuncture for pain management: molecular mechanisms of action. *Am J Chin Med*. (2020) 48:793–811. 10.1142/S0192415X20500408 32420752

[40] Qiao L Guo M Qian J Xu B Gu C Yang Y. Research advances on acupuncture analgesia. *Am J Chin Med.* (2020) 48:245–58. 10.1142/S0192415X20500135 32138535

[41] Yuan QL Wang P Liu L Sun F Cai YS Wu WT et al.. Acupuncture for musculoskeletal pain: a meta-analysis and meta-regression of sham-controlled randomized clinical trials. *Sci Rep*. (2016) 6:30675. 10.1038/srep30675 27471137 PMC4965798

[42] Wong Lit Wan D Wang Y Xue C Wang L Liang F Zheng Z. Local and distant acupuncture points stimulation for chronic musculoskeletal pain: a systematic review on the comparative effects. *Eur J Pain.* (2015) 19:1232–47. 10.1002/ejp.671 25690699

[43] Fang J Jin Z Wang Y Li K Kong J Nixon EE et al.. The salient characteristics of the central effects of acupuncture needling: limbic-paralimbic-neocortical network modulation. *Hum Brain Mapp*. (2009) 30:1196–206. 10.1002/hbm.20583 18571795 PMC6871074

[44] Gao J He L Yu X Wang L Chen H Zhao B et al.. Rehabilitation with a combination of scalp acupuncture and exercise therapy in spastic cerebral palsy. *Complement Ther Clin Pract.* (2019) 35:296–300. 10.1016/j.ctcp.2019.03.002 31003673

[45] Pei X Li Q Huang G Liao J Huang Y Chen Z et al.. Immediate efficacy of acupuncture combined with active exercise as 10 min rapid therapy for pain and movement disorders in patients suffering from acute stiff neck: protocol for a randomised controlled trial. *BMJ Open*. (2024) 14:e080793. 10.1136/bmjopen-2023-080793 39043589 PMC11268042

[46] Sun M Tao S Geng G Peng J Ma X Yan M et al.. Identification of the optimal points for the acupuncture treatment of neck pain in China: protocol for a multicenter, matched, case-control study. *BMJ Open*. (2019) 9:e029194. 10.1136/bmjopen-2019-029194 31439605 PMC6707690

[47] Dusek JA Kallenberg GA Storrow AB Hughes RM Coyne CJ Vago DR et al.. Acupuncture in the emergency department (ACUITY): results from a BraveNet multi-center feasibility randomized controlled trial. *Integr Med Res.* (2024) 13:101095. 10.1016/j.imr.2024.101095 39640074 PMC11617945

[48] Nielsen A Dyer NL Lechuga C McKee MD Dusek JA. Fidelity to the acupuncture intervention protocol in the ACUpuncture In The EmergencY department for pain management (ACUITY) trial: expanding the gold standard of STRICTA and CONSORT guidelines. *Integr Med Res*. (2024) 13:101048. 10.1016/j.imr.2024.101048 38841077 PMC11151162

[49] Nielsen A Olson J Quesada M Zhu C Raskin E Vang B et al.. Acupuncture intervention for acute pain in the emergency department trial: a consensus process. *Acupunct Med.* (2022) 40:339–46. 10.1177/09645284221076507 35229658 PMC10948001

