# Supplementary material for: Core acupoint selection strategies and multifactorial analysis for acute musculoskeletal pain
Source: Front Med (Lausanne). 2026 May 7;13:1805633. doi: 10.3389/fmed.2026.1805633 (PMC13190441; doi:10.3389/fmed.2026.1805633)
Supplement: Supplementary file 1 [file Table_1.docx]

CNKI

#1: 主题 = 针灸 + 针刺 + 取穴 + 选穴 194556

#2: 主题 = 急性肌肉骨骼痛 + 急性肌肉疼痛 + 急性疼痛 + 急性软组织损伤 + 急性运动损伤 + 扭伤 + 拉伤 + 挫伤 23792

#3: 篇名 = 慢性 + 退行性改变 + 术后 + 内脏疼痛 + 研究进展 + 小鼠 +荟萃分析 + 系统评价 + 调查 2111152

#4: (#1) AND (#2) NOT #3 2424

VIP

#1: 题名或关键词 = 针灸 + 针刺 + 取穴 + 选穴 154365

#2: 题名或关键词 = 急性肌肉骨骼痛 + 急性肌肉疼痛 + 急性疼痛 + 急性软组织损伤 + 急性运动损伤 + 扭伤 + 拉伤 + 挫伤 15642

#3: 题名 = 慢性 + 退行性改变 + 术后 + 内脏疼痛 + 研究进展 + 小鼠 +荟萃分析 + 系统评价 + 调查 2543536

#4: (#1) AND (#2) NOT #3 1764

WangFang

#1: 主题 = 针灸 OR 针刺 OR 取穴 OR 选穴 142570

#2: 主题 = 急性肌肉骨骼痛 OR 急性肌肉疼痛 OR 急性疼痛 OR 急性软组织损伤 OR 急性运动损伤 OR 扭伤 OR 拉伤 OR 挫伤 42460

#3: 题名 = 慢性 OR 退行性改变 OR 术后 OR 内脏疼痛 OR 研究进展 OR 小鼠 OR荟萃分析 OR 系统评价 OR 调查 2,728,423

#4: (#1) AND (#2) NOT #3 2,549

SinoMed

#1: 主题 = "针灸"[加权:扩展] OR "针刺"[加权:扩展] OR "取穴"[加权:扩展] OR "选穴"[加权:扩展] 142570

#2: 主题 = "急性肌肉骨骼痛"[加权:扩展] OR "急性肌肉疼痛"[加权:扩展] OR "急性疼痛"[加权:扩展] OR "急性软组织损伤"[加权:扩展] OR "急性运动损伤"[加权:扩展] OR "扭伤"[加权:扩展] OR "拉伤"[加权:扩展] OR "挫伤"[加权:扩展] 10791

#3: 中文标题 = "慢性"[中文标题:智能] OR "退行性改变"[中文标题:智能] OR "术后"[常用字段:智能] OR "内脏疼痛"[常用字段:智能] OR "研究进展"[常用字段:智能] OR "小鼠"[常用字段:智能] OR "荟萃分析"[常用字段:智能] OR "系统评价"[常用字段:智能] OR "调查"[常用字段:智能] 3296009

#4: (#1) AND (#2) NOT #3 419

WOS
#1 TS = (acupuncture OR pharmacopuncture) 24829

#2 TS = (Musculoskeletal pains OR Pain, Musculoskeletal OR Pains, Musculoskeletal OR acute pain OR Pains, Acute OR Pain, Acute OR Soft tissue injury OR Injuries, Athletic OR Athletic Injury OR Injury, Athletic OR Injuries, Sports OR Injury, Sports OR Sports Injury OR Sports Injuries OR strains and sprains OR strain OR sprain OR trauma) 1810201

#3 (#1 AND #2) NOT TS = (Chronic OR degenerative change OR postoperative OR visceral pain OR progression OR mouse OR meta OR systematic review OR investigation) and Review Article or Letter or Proceeding Paper or Editorial Material or Retracted Publication or Meeting Abstract or Reprint or News Item (Exclude – Document Types) 568

Pubmed
#1 Title/Abstract = acupuncture OR pharmacopuncture 31857

#2 Title/Abstract = (Musculoskeletal pains OR Pain, Musculoskeletal OR Pains, Musculoskeletal OR acute pain OR Pains, Acute OR Pain, Acute OR Soft tissue injury OR Injuries, Athletic OR Athletic Injury OR Injury, Athletic OR Injuries, Sports OR Injury, Sports OR Sports Injury OR Sports Injuries OR strains and sprains OR strain OR sprain OR trauma) [901563](https://pubmed.ncbi.nlm.nih.gov/?term=Musculoskeletal+pains%5BTitle%2FAbstract%5D+OR+Pain%2C+Musculoskeletal%5BTitle%2FAbstract%5D+OR+Pains%2C+Musculoskeletal%5BTitle%2FAbstract%5D+OR+acute+pain%5BTitle%2FAbstract%5D+OR+Pains%2C+Acute%5BTitle%2FAbstract%5D+OR+Pain%2C+Acute%5BTitle%2FAbstract%5D+OR+Soft+tissue+injury%5BTitle%2FAbstract%5D+OR+Injuries%2C+Athletic%5BTitle%2FAbstract%5D+OR+Athletic+Injury%5BTitle%2FAbstract%5D+OR+Injury%2C+Athletic%5BTitle%2FAbstract%5D+OR+Injuries%2C+Sports%5BTitle%2FAbstract%5D+OR+Injury%2C+Sports%5BTitle%2FAbstract%5D+OR+Sports+Injury%5BTitle%2FAbstract%5D+OR+Sports+Injuries%5BTitle%2FAbstract%5D+OR+strains%5BTitle%2FAbstract%5D+AND+sprains%5BTitle%2FAbstract%5D+OR+strain%5BTitle%2FAbstract%5D+OR+sprain%5BTitle%2FAbstract%5D+OR+trauma%5BTitle%2FAbstract%5D&sort=date)

#3 (#1 AND #2) NOT Title = (Chronic OR degenerative change OR postoperative OR visceral pain OR progression OR mouse OR meta OR systematic review OR investigation) 333

Embase

#1 'acupuncture'/exp OR acupuncture:ti,ab,kw OR shonishin:ti,ab,kw OR 'acupuncture therapy':ti,ab,kw 71,916

#2 'musculoskeletal pain'/exp OR 'locomotor pain':ti,ab,kw OR 'pain, musculoskeletal':ti,ab,kw OR 'musculoskeletal pain':ti,ab,kw
231,712

#3 'pain'/exp OR 'acute pain':ti,ab,kw OR 'deep pain':ti,ab,kw OR 'lightning pain':ti,ab,kw OR 'nocturnal pain':ti,ab,kw OR 'pain response':ti,ab,kw OR 'pain syndrome':ti,ab,kw OR 'treatment related pain':ti,ab,kw OR 'pain':ti,ab,kw 2,403,813

#4 'soft tissue injury'/exp OR 'soft tissue injuries':ti,ab,kw OR 'soft tissue trauma':ti,ab,kw OR 'soft tissue wound':ti,ab,kw OR 'soft tissue injury':ti,ab,kw 17057

#5 'sport injury'/exp OR 'athlete injury':ti,ab,kw OR 'athlete trauma':ti,ab,kw OR 'athletic injuries':ti,ab,kw OR 'athletic injury':ti,ab,kw OR 'athletic trauma':ti,ab,kw OR 'injury, athletic':ti,ab,kw OR 'sport accident':ti,ab,kw OR 'sport related injury':ti,ab,kw OR 'sport trauma':ti,ab,kw OR 'sportrelated injury':ti,ab,kw OR 'sports injury':ti,ab,kw OR 'sports related injury':ti,ab,kw OR 'sports trauma':ti,ab,kw OR 'sportsrelated injury':ti,ab,kw OR 'sport injury':ti,ab,kw 43,581

#6 'sprain'/exp OR ('sprained joints'/exp OR 'sprained joints' OR 'sprained ligament'/exp OR 'sprained ligament' OR 'sprain'/exp OR 'sprain') AND ('athlete injury':ti,ab,kw OR 'athlete trauma':ti,ab,kw OR 'athletic injuries':ti,ab,kw OR 'athletic injury':ti,ab,kw OR 'athletic trauma':ti,ab,kw OR 'injury, athletic':ti,ab,kw OR 'sport accident':ti,ab,kw OR 'sport related injury':ti,ab,kw OR 'sport trauma':ti,ab,kw OR 'sportrelated injury':ti,ab,kw OR 'sports injury':ti,ab,kw OR 'sports related injury':ti,ab,kw OR 'sports trauma':ti,ab,kw OR 'sportsrelated injury':ti,ab,kw OR 'sport injury':ti,ab,kw) 8,374

#7 #2 OR #3 OR #4 OR #5 OR #6 2,455,486

#8 chronic:ti OR 'degenerative change':ti OR postoperative:ti OR 'visceral pain':ti OR progression:ti OR mouse:ti OR meta:ti OR 'systematic review':ti OR 'experimental therapy':ti 1,849,805

#9 #1 AND #7 NOT #8 1732

#10 #9 AND ('case control study'/de OR 'case report'/de OR 'case study'/de OR 'clinical article'/de OR 'clinical audit'/de OR 'clinical study'/de OR 'clinical trial'/de OR 'clinical trial topic'/de OR 'comparative effectiveness'/de OR 'controlled clinical trial'/de OR 'controlled study'/de OR 'major clinical study'/de OR 'multicenter study'/de OR 'multicenter study topic'/de OR 'observational study'/de OR 'prospective study'/de OR 'randomized controlled trial'/de OR 'randomized controlled trial topic'/de) 1169

Cochrane

#1 MEacupuncture OR pharmacopuncture 24361

#2 Musculoskeletal pains OR Pain, Musculoskeletal OR Pains, Musculoskeletal OR acute pain OR Pains, Acute OR Pain, Acute OR Soft tissue injury OR Injuries, Athletic OR Athletic Injury OR Injury, Athletic OR Injuries, Sports OR Injury, Sports OR Sports Injury OR Sports Injuries OR strains and sprains OR strain OR sprain OR trauma 87269

#3 Chronic OR degenerative change OR postoperative OR visceral pain OR progression OR mouse OR meta OR systematic review OR investigation 543860

#4 ((#1 and #2 ) not #3 ) 445
