# Supplementary material for: Core acupoint selection strategies and multifactorial analysis for acute musculoskeletal pain
Source: Front Med (Lausanne). 2026 May 7;13:1805633. doi: 10.3389/fmed.2026.1805633 (PMC13190441; doi:10.3389/fmed.2026.1805633)
Supplement: Supplementary file 2 [file Table_2.docx]

| **Acupoint (Chinese)** | **Acupoint (English)** | **Type** | **Location** |
| --- | --- | --- | --- |
| 腰痛点 | EX-UE7 | Hand Acupuncture | Midpoint between the 2nd and 3rd, 4th and 5th metacarpal bones on the dorsum of the hand, in the depression radial to the tendon of the extensor indicis muscle |
| 肘穴区 | AP-SF3 | Auricular Acupuncture | Below the wrist zone, located at the auricular scaphoid area zone 3 |
| 肩穴区 | AP-SF4,5 | Auricular Acupuncture | Below the elbow area, i.e., the scapha areas 4 and 5 |
| 肺穴区 | AP-CO14 | Auricular Acupuncture | Located around the heart and trachea regions of the ear, at the 14th area of the auricular concha |
| 跗骨窦 | EX-Fugudou | Foot Acupuncture | A conical cavity between the neck of the talus and the anterior-superior aspect of the calcaneus |
| 上5穴 | WAA-Up 5 | Wrist-Ankle Acupuncture | Corresponding to Waiguan (TE5) |
| 踝穴区 | AP-AH3 | Auricular Acupuncture | Below the toe and heel zones, located at the lower half of the upper third of the superior crus of the antihelix |
| 上4穴 | WAA-Up 4 | Wrist-Ankle Acupuncture | On the radial side of the dorsal wrist, 2 finger breadths above the transverse wrist crease, near the radial border |
| 腕穴区 | AP-SF2 | Auricular Acupuncture | Below the finger zone, located at the auricular scaphoid area zone 2 |
| 风溪穴区 | AP-SF1，2i | Auricular Acupuncture | In front of the auricular tubercle, between the finger zone and the wrist zone |
| 下焦穴 | EX-Xiajiao | Body Acupuncture | On the line connecting the midpoint of the tragus and ST6 (Jiache), at the 3/4 point downward |
| 挫闪穴 | EX-Cuoshan | Body Acupuncture | At the 3/4 point on the line connecting Yangchi (TE4) and the lateral epicondyle of the humerus |
| 池下穴 | EX-Chixia | Body Acupuncture | Midpoint between Quchi (LI11) and Shousanli (LI10) |
| 痛灵穴 | EX-Tongling | Body Acupuncture | On the dorsum of the hand, between the 3rd and 4th metacarpal bones, 0.5 cun proximal to the metacarpophalangeal joint |
| 重子穴 | EX-Chongzi | Hand Acupuncture | Located one cun below the tiger's mouth on the palm, close to the medial side of the metacarpal bone of the index finger |
| 灵骨穴 | EX-Linggu | Hand Acupuncture | Located at the junction of the 1st and 2nd metacarpal bones on the back of the hand |
| 小节穴 | EX-Xiaojie | Hand Acupuncture | Located beside the metacarpal bone at the first metacarpophalangeal joint |
| 中平穴 | EX-Zhongping | Body Acupuncture | Located on the anterior edge of the fibula on the lateral side of the lower leg |
| 肩前穴 | EX-Jianqian | Body Acupuncture | Located on the anterior part of the shoulder, 1.5 cun directly above the anterior axillary fold |
| 眼部膀胱区穴 | OA-2 | Eye Acupuncture | Eye acupuncture area 2 |
| 腹部压痛点 | EX-Ashi point | Body Acupuncture | Tender point in the abdomen |
| 面部腰穴 | EX-Face Yao | Body Acupuncture | At the junction of the lower edge of the intertragic notch and the line to the mandibular angle, at the anterior 0.5 cun |
| 颈根穴 | EX-Jinggen | Body Acupuncture | Located at the depression between the clavicular head and the sternal head of the sternocleidomastoid muscle, on the upper edge of the medial end of the clavicle |
| 腰突穴 | EX-Yaotu | Body Acupuncture | 1 cm lateral to the lumbar spine at the painful spot |
| 腰痛穴 | EX-Yaotong | Scalp Acupuncture | 1.5 cun directly above Yintang |
| 下闪电穴 | EX-Xiashandian | Body Acupuncture | 6 cun lateral to the tip of the coccyx |
| 腰骶椎穴区 | AP-AH9 | Auricular Acupuncture | Located at the upper 2/5 of the antihelix |
| 膝痛穴 | EX-Xitong | Body Acupuncture | At the center of the lateral end of the cubital crease |
| 臀痛穴 | EX-Tuntong | Body Acupuncture | Midpoint between the posterior axillary fold and the acromion |
| 阑尾穴 | EX-LE7 | Body Acupuncture | 2 cun below ST36 |
| 健侧足运感区 | Contralateral Foot Movement Sensation Area | Scalp Acupuncture | 1 cm lateral to the midpoint of the anterior–posterior midline of the head, 3 cm parallel line posteriorly |
| 臀穴区 | AP-AH7 | Auricular Acupuncture | At the posterior 1/3 of the inferior crus of the antihelix area 7 |
| 坐骨神经穴区 | AP-AH6 | Auricular Acupuncture | At the anterior 2/3 of the inferior crus of the antihelix area 6 |
| 交感穴区 | AP-AH6a | Auricular Acupuncture | At the junction of the anterior end of the inferior crus of the antihelix and the inner margin of the helix, i.e., the anterior end of the antihelix area 6 |
| 耳尖穴 | AP-HX6,7i | Auricular Acupuncture | Located at the upper tip where the auricle is folded forward, i.e., at the junction of the 6th and 7th areas of the helix |
| 肩痛穴 | EX-Jiantong | Body Acupuncture | 2 cun below ST36, slightly lateral toward the fibula |
| 下5穴 | WAA-Down 5 | Wrist-Ankle Acupuncture | On the lateral side, along the posterior border of the fibula, 3 finger breadths above the highest point of the lateral malleolus |
| 下6穴 | WAA-Down 6 | Wrist-Ankle Acupuncture | Along the lateral border of the Achilles tendon, 3 finger breadths above the highest point of the lateral malleolus |
| 下2穴 | WAA-Down 2 | Wrist-Ankle Acupuncture | On the medial side, along the posterior border of the tibia, 3 finger breadths above the highest point of the medial malleolus |
| 下3穴 | WAA-Down 3 | Wrist-Ankle Acupuncture | 1 fen inward from the anterior border of the tibia |
| 下4穴 | WAA-Down 4 | Wrist-Ankle Acupuncture | Located at the midpoint of the tibialis anterior muscle, between the anterior border of the tibia and the anterior margin of the fibula |
| 阿是穴 | Ashi Point | Body Acupuncture/ Scalp Acupuncture/ Wrist-Ankle Acupuncture | Tender point at the site of pain |
| 对侧阿是穴 | EX-Ashi Point | Body Acupuncture/Wrist-Ankle Acupuncture | Tender point at the corresponding site on the opposite side |
| 扭伤穴 | EX-Niushang | Body Acupuncture | The junction between the upper one-fourth and the lower three-fourths of the line connecting Yangchi (TE4) and Quchi (LI11) |
| 夹脊穴 | EX-B2 | Body Acupuncture | Located in the spinal region, on both sides below the spinous processes of the 1st thoracic vertebra to the 5th lumbar vertebra, 0.5 cun away from the posterior midline |
| 颈夹脊穴 | EX-Jingjiaji | Body Acupuncture | Located 0.5 cun lateral to the inferior aspect of the spinous processes of the cervical vertebrae |
| 带脉后 | EX-Daimaihou | Body Acupuncture | Located posterior to the Daimai (GB26) point |
| 五虎穴 | EX-Wuhu | Hand Acupuncture | Located at the boundary between the red and white skin on the radial side of the first phalanx of the thumb |
| 额中穴 | EX-Ezhong | Scalp Acupuncture | Located on the midline of the head, 1 cun directly above the glabella |
| 心门穴 | EX-Xinmen | Body Acupuncture | Located on the medial side of the forearm, 1.5 cun below the cubital crease |
| 人字缝尖 | SA-Renzifengjian | Scalp Acupuncture | Located between the occipital bone and the parietal bone in the skull |
| 星点 | SA-Xingdian | Scalp Acupuncture | Located at the junction of the lambdoid suture and the sagittal suture, approximately 6 centimeters above the external occipital protuberance |
| 翼点 | SA-Yidian | Scalp Acupuncture | Located approximately 3.5 cm posterior to the lateral canthus of the palpebral fissure, and 4 cm away from the upper edge of the zygomatic arch |
| 顶颞后斜线 | SA-MS7 | Scalp Acupuncture | Located on the lateral side of the head |
| 十七椎 | EX-B8 | Body Acupuncture | Located on the midline of the human lumbar region, in the depression below the spinous process of the 5th lumbar vertebra |
| 臀中穴 | EX-Tunzhong | Body Acupuncture | Located in the buttock region, at the depression below the iliac crest, approximately near the midpoint of the line connecting the anterior superior iliac spine and the greater trochanter of the femur |
| 中泉穴 | EX-UE3 | Hand Acupuncture | In the dorsal transverse crease of the wrist, within the depression on the radial side of the extensor tendons of the fingers |
